# An assessment of dynamic facial emotion recognition and theory of mind in children with ADHD: An eye-tracking study

**DOI:** 10.1371/journal.pone.0298468

**Published:** 2024-02-08

**Authors:** Abdullah Bozkurt, Esen Yıldırım Demirdöğen, Müberra Kolak Çelik, Mehmet Akif Akıncı

**Affiliations:** Department of Child and Adolescent Psychiatry, Ataturk University, Erzurum, Türkiye; University Ceuma, BRAZIL

## Abstract

Deficits in social cognition in attention deficit hyperactivity disorder (ADHD) have been associated with difficulties in functioning. Since recognizing emotional facial expressions is essential for developing the perceptual components of the theory of mind (ToM), it is important to assess this relationship in children with ADHD. This study therefore compared the recognition of emotional stimuli and gaze patterns between children with ADHD and healthy children using eye-tracking with dynamic facial images. It also examined the relationship between facial emotion recognition accuracy, gaze patterns, ToM scores, and ADHD symptoms. Children with ADHD aged 8–13 (n = 47) and a control group (n = 38) completed a facial emotion recognition test, ToM tests, and the Conners’ Parent Rating Scale. Participants’ gaze patterns in response to dynamic facial emotion expressions were recorded using eye-tracking technology. Children with ADHD exhibited significantly lower accuracy in the recognition of the facial expressions of disgust and anger. The percentage fixation in the eye region was also significantly lower for happy, angry, sad, disgusted, and neutral emotions in the children with ADHD compared to the control group. No relationship was determined between the percentage of fixations on facial areas of interests and ADHD symptoms or ToM tests. This study provides evidence that children with ADHD experience deficits in visual attention to emotional cues. In addition, it suggests that facial emotion recognition deficits in children with ADHD represent a separate domain of social cognition that develops independently of ToM skills and core symptoms. Understanding and treating the social difficulties of individuals with ADHD may help improve their social functioning.

## 1. Introduction

Attention deficit hyperactivity disorder (ADHD) is a neurodevelopmental disorder characterized by inattention, hyperactivity, and impulsivity, resulting in impairments in social, educational, and familial functioning [1]. Children with ADHD frequently exhibit deficits in social competence, including poor eye contact, inappropriate interpersonal relationships, and difficulty in building peer relationships [2,3]. Impairment in social cognitive skills in ADHD is likely to contribute to deficits in social skills and to impaired functioning. Social cognition is a broad field in which different cognitive abilities are used to process social information and achieve success in social situations. Recognition of emotional expressions (often measured using still image/drawing sets or registered vocal expressions of six basic emotions) and theory of mind (ToM), the capacity to assess mental states (emotions, beliefs, intentions and desires) in others and to understand and predict the behavior of others based on their mental states, are the most important areas of social cognition [4]. Research has shown that children diagnosed with ADHD also experience social cognition deficits [4,5].

Emotional facial expressions are a non-verbal tool for communicating emotions and recognizing the emotional states of others [6]. Recognition of emotional facial expressions represents the capacity to identify, differentiate, and categorize emotional states based on facial expressions. This plays an important role in social functions, such as peer relationships and responding to vivid emotional situations [7]. Difficulties in the recognition of emotional facial expression may derive from various mechanisms associated with cognitive impairments, deficits in the processing of social information, specific changes in the brain systems underlying facial processing abilities, or comorbid conditions [8]. Recent years have seen a growing body of research into the recognition of facial emotion, which plays a key role in the development of social cognition in ADHD since the disorder progresses with cognitive impairments. Although some studies have observed impairment in the recognition of facial emotion in ADHD, others have found no such disturbance. However, the majority of these studies did not involve an analysis of specific emotion types. The few studies investigating specific disorders reported that fear was the most impaired facial expression in ADHD, with compromises also being reported in the recognition of angry, sad, disgusted, happy, and neutral faces [9]. However, previous findings were based on photographs showing static, full-blown emotions. This procedure may lack sufficient sensitivity in terms of noticing very slight differences in emotion recognition and may lead to inconsistencies in the specificity of the emotion recognition deficit [10].

The recognition of emotional stimuli is based on orientation toward these and maintaining attention. Researchers suggest that inattention in ADHD represents an essential factor in emotion recognition deficits by causing important clues in emotional stimuli to be missed [11]. However, there are insufficient data to suggest that attention difficulties in children with ADHD affect the perception of emotional facial expressions. Eye-tracking technology in emotion recognition has provided useful information for examining the tracking patterns of emotional stimuli and visual inattention in recent years [12]. The results of eye tracking show where the child is looking when examining an image, while recognition of emotional facial expressions results indicate how the child is using that information. A combination of these two methods yields indicators of information input and output. In order to understand the recognition of emotional facial expressions deficits in children with ADHD, it is crucially important to determine whether and for how long they look at essential cues. The limited numbers of previous eye-tracking studies in this area have reported that young people with ADHD are less likely to look at important cues in imaging patterns and take longer to recognize emotions [11,13]. However, as described above, previous findings were based on photographs showing static, full-blown emotions. Examining the ability to detect subtle emotional expressions that can be assessed in dynamic images can better identify deficits in real-life social interactions in which facial expressions change rapidly. This can be achieved through a series of emotion recognition paradigms, in which the object’s facial expressions slowly change from neutral to fully developed emotion [10]. This methodology permits a more sensitive and ecologically reliable evaluation of emotional and social perceptual thresholds in ADHD.

ToM is thought to represent a high-level process in the social cognition system involving perceptual, emotional, and cognitive processing. It includes both cognitive and affective components. Cognitive ToM relates to conclusions regarding the ideas and intentions of others, while emotional ToM refers to inferences concerning the emotions and feelings of others (assessed using the Reading the Mind in the Eyes and Faux Pas Recognition Task) [14,15]. The neural mechanisms that support the basic sensory processing of social information and the ToM system are thought to exhibit an interactive, bidirectional relationship [16]. In this context, the recognition of emotional facial expressions is also essential for the maturation of the perceptual elements of ToM [17]. It is essential to investigate whether social cognitive impairments in ADHD, in areas such as emotion recognition and theory of mind, are an independent abnormality or exist secondary to mental abnormalities in the disorder because neurocognitive abilities contribute to the performance on social cognitive tasks of patients with neuropsychiatric disorders [18]. The relationship between facial emotion recognition skills and ToM and symptoms may represent a useful guide concerning where to intervene in order to address social deficits in children with ADHD. Interventions can direct children’s attention to the proper orientations of faces in case of deficiencies in the most fundamental class of emotion recognition (such as the eyes and mouth). If children with ADHD do not exhibit deficits at the primary level, and if their viewing patterns are similar to those of healthy children, interventions can target higher-level social cognitive skills (such as sign interpretation, irony, metaphor, and implication understanding) that contribute to emotion recognition deficits. To the best of our knowledge, no previous studies have used an eye-tracking device to examine dynamic facial images in children with ADHD and assessed their relationship with higher-order social cognitive processes and symptoms.

Specific research into this important topic is inadequate, despite several reports of emotion recognition deficits. Our hypothesis in this study was that children with ADHD may exhibit deficits in emotional facial recognition and attention to emotional cues, which may represent a social cognition deficit unrelated to the main symptoms and other skills. This study therefore 1) compared the recognition of emotional stimuli and gaze patterns between children with ADHD and healthy children using an eye-tracking method with dynamic facial emotion images, and 2) examined the relationships between emotional facial recognition accuracy, gaze patterns, ToM scores, and ADHD symptoms.

## 2. Method

### 2.1. Participants

This study was conducted in the Atatürk University Faculty of Medicine Department of Child and Adolescent Psychiatry, Türkiye, between November 1, 2022 and March 10, 2023. Clinical evaluations were performed using the Diagnostic and Statistical Manual of Mental Disorders-5 (DSM-5) to identify children with ADHD [1]. Diagnoses were confirmed using the Schedule for Affective Disorders and Schizophrenia for School-Age Children-Present and Lifetime Version (K-SADS-PL) [19]. The control group consisted of randomly selected children from a local school with no psychiatric disorders according to K-SADS-PL. The inclusion criteria for children in the ADHD group were being newly diagnosed with ADHD, age 8–13 years, IQ of 79 or higher, and parental consent to participation. Exclusion criteria were the presence of mental disability, specific learning disorder, autism, any cognitive impairment or psychiatric disorder, any other concomitant medical illness or receipt of psychotropic medication, significant visual impairment at eye screening at school, and color blindness (determined using Ishihara’s color blindness test). The inclusion criteria for the healthy children were age 8–13 years, absence of any previous or current diagnosis of any psychiatric disorder, no current diagnosis of any medical illness, no significant visual impairment and no color blindness at eye screening, and parental consent to participation. Following application of the inclusion and exclusion criteria, 47 children diagnosed with ADHD and 38 healthy individuals were eventually enrolled in the study.

The study protocol was reviewed and approved by the Ataturk University Faculty of Medicine clinical research ethical committee, (B.30.2.ATA.0.01.00/669). The study was performed in compliance with the principles of the Declaration of Helsinki. Written informed consent was received from all participants and their parents.

### 2.2. Measures

#### 2.2.1. ADHD symptoms

The Conners’ Parent Rating Scale–Revised: Short Form (CPRS-R: S) is frequently used for the measurement of ADHD symptoms in children. The parent is asked to assess to what extent each of 27 items has represented a problem for the child using a four-point Likert-type scale ranging from 0 (not at all true) to 3 (very true) [20]. It consists of four subscales: oppositional, hyperactive, inattentive, and ADHD index. The Turkish-language version of the CPRS-R:S has been confirmed as valid and reliable for the Turkish population [21].

#### 2.2.2. ToM tests

*Reading the Mind in the Eyes Test-Child Version (RMET-C)*. This advanced ToM test assesses the individual’s ability to make inferences concerning another’s mental state simply by looking at eye photographs. Our participants were shown pictures of the eye area accompanied by four words describing different feelings. They were then asked to select the emotion best reflected by the eyes in the images [22]. The test has been found to discriminate between clinical and control groups and to possess high test-retest reliability and validity with other measures of social cognition [23]. The Turkish-language version was used in the present study [24].

*Faux Pas Recognition Test-Child Version (FPRT-C)*. Baron-Cohen et al. developed the Faux Pas Test for the purpose of assessing higher mental attributions [25]. Recognizing a faux pas is widely regarded as the most difficult developmental skill and as a sensitive evaluation instrument for the ToM. A faux pas refers to an individual saying something he should not have said without his being aware of having done so. It is vitally important to depict two mental states to determine when a faux pas has been committed. This ability involves both cognitive and emotional empathy components [26,27]. After listening to a narrative, the children answer four comprehension questions. In order to identify a faux pas, the child must correctly respond to all inquiries, answer a comprehension question, and understand that the faux pas resulted from a faulty belief. In the control stories, the child must determine that no faux pas occurred. Any of these questions being answered incorrectly will result in a score of zero for that particular story. The minimum possible score for the whole test is 0, and the maximum possible score is 20, with 0–10 points being awarded for the faux pas stories and 0–10 for the control stories. The Turkish version was used in the present study [28].

#### 2.2.3. Assessment of facial emotion recognition

The dynamic images used in the study were taken from the *FACES database* developed at the *Max Planck Institute for Human Development in Berlin*, *Germany*. The dynamic FACES database consists of formatted videos of young, middle-aged, and older adults with six natural emotional facial expressions, representing neutrality, sadness, disgust, fear, anger, and happiness. The FACES database provided static pictures for morphing. Videos were produced by changing from a still, neutral image to the target emotion. Each video was two seconds long (one second for the morph followed by one second for static exhibition of the expression) [29]. In the present study, each of the six basic emotions selected from the younger age group was shown six times, with each participant thus viewing a total of 36 dynamic images.

#### 2.2.4. Eye-tracking

**Fig 1:**
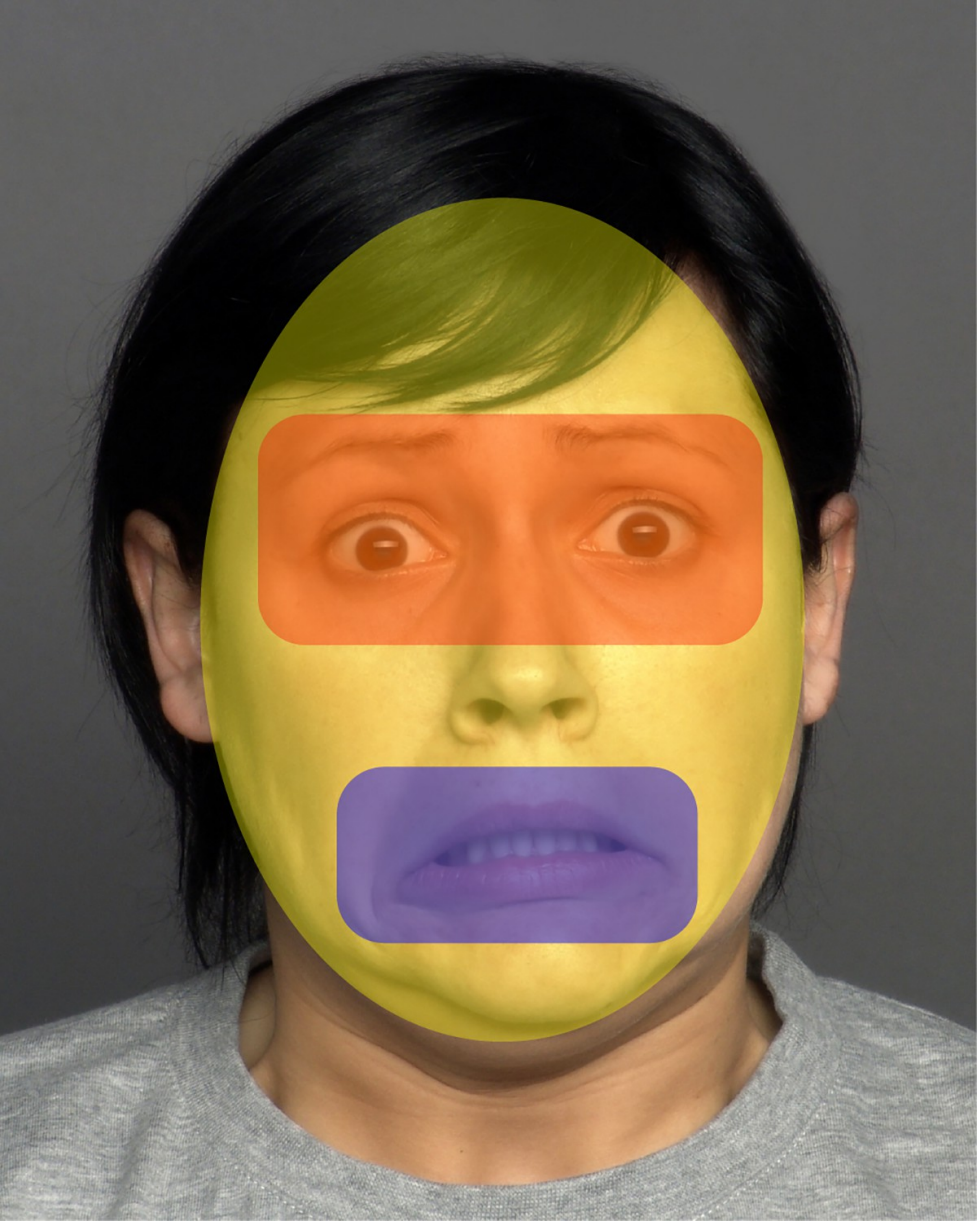
Two areas of interest in facial emotion expression: The eyes and mouth.

A nine-point calibration was performed using a computer screen at a distance of 60–65 cm from the participants. The calibration accuracy was examined, and the procedure was repeated as necessary. Facial stimuli were administered immediately after calibration. Eye movements were recorded using an SMI RED250 eye-tracker. Dynamic emotional images were shown using Experiment Center software. Eye movement data were measured using Be Gaze software, which also permits the identification of areas of interest in the visual stimulus presented. Two areas of interest (AOIs), the eyes and mouth, were identified for each stimulus image in order to assess the length of time the participants gazed at each particular area of the face (Fig 1). The parameter based on eye-tracking was the percentage of the total fixation time in all AOIs (total AOI fixation duration) divided by the total duration of all fixations on each image shown. Several previous studies have verified these measurements using eye-tracking to quantify visual attention [30].

### 2.3. Procedure

Children and their parents provided written consent to participation in the study. Once the participants’ diagnoses had been evaluated, comorbidities were assessed using K-SADS-PL. The participants were administered WISC-R and participated in the evaluation of verbal, performance, and total intelligence scores. Next, ToM tests were administered to all participants, and CPRS-R:S was applied to the ADHD group.

**Fig 2:**
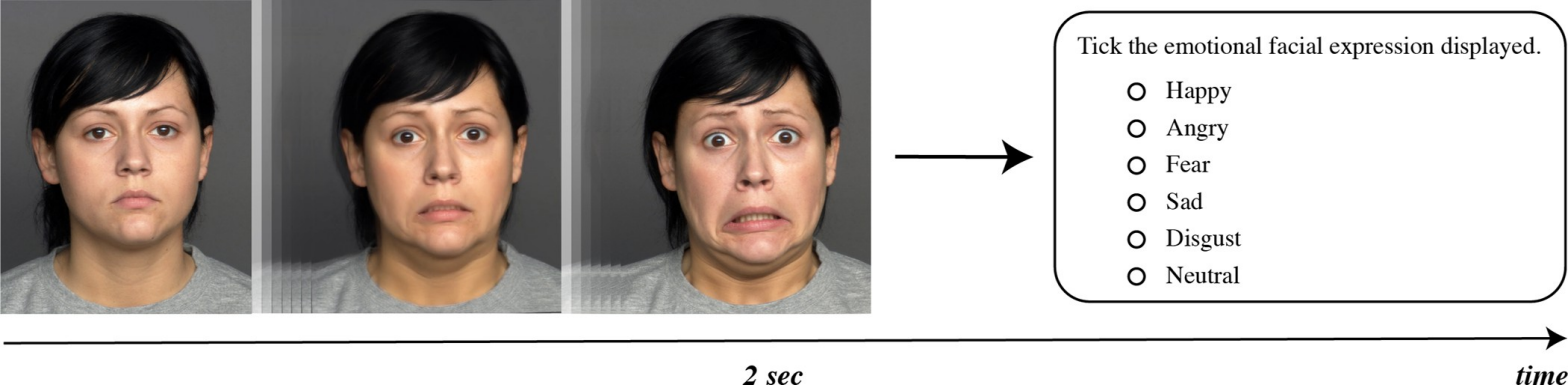
An example of facial emotion recognition task.

The children were first informed about the procedure and then taken to the laboratory where the evaluation would take place. Calibration and validation procedures were performed before the emotional images were displayed. A training trial was conducted before the emotion recognition and eye-tracking procedures. The dynamic image used changed from an initially neutral facial expression to a specific emotional facial expression within two seconds. At the end of the dynamic image, the participants were presented with a screen displaying the response options for the emotion they were watching (Fig 2). The children identified the emotions by indicating their responses on the computer screen. Thirty-six dynamic emotion images were displayed, and eye-tracking data were recorded. The evaluator allowed breaks to be taken when requested or whenever this deemed appropriate to control the effects of fatigue, maintain motivation, and reduce anxiety.

### 2.4. Statistical analysis

In the sample study, the effect size (Cohen’s d) was calculated as 0.674, with 80% power and a 95% confidence interval, indicating that 72 patients, at least 36 in each group, should be included in the study [31]. The collected data were analyzed on SPSS version 26.0 software (SPSS Inc., Chicago, IL, USA). Descriptive statistics were applied for sociodemographic data. Normality of distribution of the variables was assessed using the Shapiro-Wilk test. Parametric methods were used for normally distributed variables. The differences between the two groups were evaluated using Student’s t test, and associations were determined by means of chi-square tests. The effect size was computed by dividing the difference between the means of the participants with ADHD and the healthy individuals by the standard deviation of the pooled within-group data. Pearson’s test was used to calculate correlation coefficients and significance between two normally distributed parameters. Spearman’s test was used to investigate the correlation coefficients and significance of non-normally distributed parameters. p values < 0.05 were regarded as statistically significant for the purposes of this study.

## 3. Results

The demographic and clinical characteristics of the study groups are presented in Table 1. Eighty-five children were enrolled in the study, 47 with ADHD and 38 healthy controls. No sex or age difference was observed between the groups. The ADHD (100.5 ± 18.9) and control (103.2 ± 18.1) groups were also matched in terms of IQ.

**Table 1:** Characteristics of the study groups.

		Groups^1^			Statistics		
Parameter^2^		ADHD	Control		*t* or *χ*^*2*^	*P*	Effect Size
Age (years)		10.0 ± 1.7	10.6 ± 1.8		1.52	0.146	0.320
Gender (male/female)		38/9	28/10		0.62	0.430	0.430
WISC-R							
Verbal IQ		98.7 ± 17.3	102.8 ± 12.0		0.49	0.622	0.275
Performance IQ		101.2 ± 18.9	103.6 ± 23.7		0.25	0.802	0.111
Total IQ		100.5 ± 18.9	103.2 ± 18.1		0.30	0.762	0.145
Emotion Facial Recognition Accuracy							
Happy		5.8 ± 0.6	5.9 ± 0.4		0.22	0.821	0.196
Angry		4.6 ± 1.0	5.2 ± 0.8		3.06	0.003	0.662
Fearful		4.6 ± 1.5	4.9 ± 1.4		0.87	0.384	0.206
Sad		4.3 ± 1.4	4.0 ± 1.6		0.79	0.431	0.199
Disgusted		4.1 ± 1.8	5.5 ± 0.8		4.16	0.000	1.005
Neutral		4.9 ± 1.5	5.4 ± 0.9		1.70	0.091	0.404
Total		28.6 ± 4.1	31.0 ± 3.3		2.86	0.005	0.644
ToM Test							
RMET-C		15.9 ± 4.3	18.5 ± 3.7		2.71	0.008	0.648
FPRT-C		12.8 ± 2.4	14.1 ± 1.9		2.48	0.015	0.600

^1^ADHD: Attention Deficit Hyperactivity Disorder Spectrum; Control: Healthy subjects. Data are expressed as mean ± standard deviation.

^2^WISC-R: Wechsler Intelligence Scale for Children-Revised; ToM: Theory of Mind; RMET-C: Reading the Mind in the Eyes Test-Child Version; FPRT-C: Faux Pas Recognition Test-Child Version.

Comparison of the two groups’ facial emotion recognition accuracy variables revealed that children with ADHD performed worse in identifying anger and disgust. No differences were found between the two groups in terms of recognition of happy, fearful, sad, or neutral emotional faces. The ADHD group scored lower on the ToM tests than the healthy controls (Table 1).

The percentage of fixation on the eye region (PFER) was significantly lower in the ADHD group than in the control group in happy, angry, sad, disgusted, and neutral emotions. The percentage of fixation on the total (eye + mouth) region (PFTR) and the percentage of fixation on the mouth region (PFMR) were not significantly different between the groups (Table 2).

**Table 2:** A comparison of the percentages of fixations on the eye, mouth, and total region out of all fixations on the face.

	Groups^1^		Statistics		
Parameter^2^	ADHD	Control	*t*	*p*	Effect Size
Happy					
PFER	32.9 ± 16.1	45.4 ± 18.3	3.33	0.001	0.725
PFMR	37.3 ± 17.9	29.9 ± 17.4	1.88	0.630	0.419
PFTR	70.2 ± 17.5	75.4 ± 17.2	1.36	0.175	0.299
Angry					
PFER	36.3 ± 15.6	46.5 ± 18.8	2.70	0.008	0.590
PFMR	32.5 ± 15.2	27.7 ± 17.7	1.32	0.188	0.290
PFTR	68.9 ± 17.2	74.3 ± 15.5	1.48	0.141	0.329
Fear					
PFER	44.7 ± 14.7	52.0 ± 20.1	1.93	0.057	0.414
PFMR	29.3 ± 13.6	24.1 ± 16.2	1.59	0.114	0.347
PFTR	74.0 ± 14.0	76.2 ±17.0	0.63	0.528	0.141
Sad					
PFER	43.4 ± 18.3	55.6 ± 19.7	2.91	0.005	0.641
PFMR	26.3 ± 16.1	21.7 ± 19.7	1.33	0.185	0.255
PFTR	69.8 ± 19.0	77.4 ± 15.2	1.97	0.052	0.441
Disgusted					
PFER	39.1 ± 17.4	50.4 ± 21.5	2.66	0.009	0.577
PFMR	31.9 ± 15.6	26.7 ± 18.1	1.39	0.167	0.307
PFTR	71.1 ± 18.3	77.2 ± 14.2	1.68	0.096	0.372
Neutral					
PFER	45.9 ± 16.3	57.6 ± 19.5	2.98	0.004	0.651
PFMR	26.7 ± 16.8	21.4 ± 16.6	1.44	0.153	0.317
PFTR	72.7 ± 17.6	79.0 ± 15.6	1.73	0.087	0.378

^1^ADHD: Attention Deficit Hyperactivity Disorder Spectrum; Control: Healthy subjects. Data are mean ± standard deviation.

^2^PFER:Percentage of Fixation on the Eye Region; PFMR: Percentage of Fixation on the Mouth Region; PFMR: Percentage of Fixation on the Total Region.

The correlation values between facial emotion recognition accuracy and PFER for the same facial expression in the ADHD group were happy r = 0.12 p = 0.39, angry r = -0.03 p = 0.81, fearful r = 0.16 p = 0.36, sad, r = 0.04 p = 0.75, disgusted r = -0.14 p = 0.34, and neutral r = -0.09 p = 0.52. The correlation values between facial emotion recognition accuracy and PFTR for the same facial expression in the ADHD group were happy r = -0.22 p = 12, angry r = -0.20 p = 0.17, fearful r = -0.08 p = 0.58, sad r = -0.03 p = 05, disgusted r = -0.07 p = 0.62, and neutral r = -0.13 p = 0.38. No significant correlation was found.

In the ADHD group, the CPRS-R: S subscales oppositional and hyperactivity-impulsivity and ADHD index scores were correlated with accuracy for the sad parameter. A positive correlation was also found between the ToM test RMET-C, FPRT-C, and accuracy in the disgust parameter. The relationships between facial emotion recognition accuracy and the CPRS-R: S subscales and ToM tests are shown in Table 3. No significant correlation was found in the ADHD group between the percentage of fixation on areas of interest in the face and the CPRS-R:S subscale scores and ToM tests (Table 4).

**Table 3:** Correlations between facial emotional recognition accuracy and CPRS-R: S subscales and ToM tests in the ADHD group.

		ADHD^1^ group Emotional Facial Recognition Accuracy					
Parameter^2^		Happy	Angry	Fearful	Sad	Disgusted	Neutral
CPRS-R: S							
Oppositional	r *p*	-0.07 0.62	0.21 0.14	-0.13 0.38	0.34* 0.01	-0.19 0.18	-0.14 0.34
Inattention	r *p*	-0.11 0.45	0.18 0.21	-0.01 0.90	0.15 0.29	0.12 0.42	0.21 0.14
Hyperactivity—Impulsivity	r *p*	-0.15 0.30	0.25 0.07	-0.02 0.89	0.32 0.02*	-0.05 0.72	-0.03 0.82
ADHD Index	r *p*	-0.13 0.37	0.00 0.95	-0.07 0.59	0.37 0.01*	-0.14 0.33	0.03 0.80
ToM tests							
RMET-C	r *p*	0.01 0.98	0.10 0.94	0.05 0.71	-0.26 0.07	0.40 0.01*	0.05 0.74
FPRT-C	r *p*	0.04 0.98	-0.09 0.53	-0.05 0.72	-0.27 0.06	0.30 0.03*	0.05 0.70

^1^ADHD: Attention Deficit Hyperactivity Disorder.

^2^ CPRS-R: S: Conners’ Parent Rating Scale–Revised: Short Form; ToM: Theory of Mind; RMET-C: Reading the Mind in the Eyes Test-Child Version; FPRT-C: Faux Pas Recognition Test-Child Version.

*p < 0.05.

**Table 4:** Correlation of the percentage of fixation of the face to the areas of interest in the ADHD group between the CPRS-R: S subscales and the correlation between the ToM tests.

		CPRS-R: S^1^				ToM Tests^2^	
Parameter^3^		Oppositional	Inattention	Hyperactivity—Impulsivity	ADHD Index	RMET-C	FPRT-C
Happy							
PFER	r *p*	-0.03 0.82	0.03 0.82	-0.03 0.82	0.02 0.86	0.27 0.63	0.03 0.81
PFTR	r *p*	-0.20 0.16	0.03 0.82	-0.11 0.42	-0.11 0.42	0.08 0.58	-0.01 0.97
Angry							
PFER	r *p*	-0.07 0.59	0.02 0.86	-0.06 0.68	0.01 0.93	0.21 0.14	-0.13 0.38
PFTR	r *p*	-0.32 0.02	-0.08 0.55	-0.27 0.05	-0.15 0.31	0.13 0.36	0.01 0.93
Fearful							
PFER	r *p*	0.09 0.52	0.12 0.42	0.02 0.84	0.09 0.54	0.17 0.25	0.06 0.67
PFTR	r *p*	-0.23 0.11	-0.04 0.75	-0.27 0.06	-0.08 0.57	0.11 0.44	0.08 0.56
Sad							
PFER	r *p*	0.03 0.79	0.14 0.33	0.06 0.66	0.16 0.25	0.13 0.39	-0.08 0.59
PFTR	r *p*	-0.20 0.17	0.10 0.49	-0.08 0.58	0.09 0.52	-0.01 0.91	-0.12 0.39
Disgusted							
PFER	r *p*	0.11 0.44	0.30* 0.03	0.05 0.72	0.27 0.06	0.10 0.49	-0.12 0.43
PFTR	r *p*	-0.13 0.38	0.13 0.35	-0.13 0.35	0.12 0.40	-0.04 0.77	-0.18 0.23
Neutral							
PFER	r *p*	0.18 0.21	0.16 0.26	0.14 0.32	0.12 0.39	-0.06 0.69	-0.12 0.41
PFTR	r *p*	-0.18 0.22	0.08 0.57	-0.14 0.31	0.06 0.67	-0.25 0.09	-0.25 0.09

^1^ CPRS-R: S: Conners’ Parent Rating Scale–Revised: Short Form.

^2^ ToM: Theory of Mind; RMET-C: Reading the Mind in the Eyes Test-Child Version; FPRT-C: Faux Pas Recognition Test-Child Version.

^3^ PFER: Percentage of Fixation on the Eye Region; PFMR: Percentage of Fixation on the Total Region.

## 4. Discussion

Few studies to date have examined patterns of visual attention during facial emotional recognition in children with ADHD. The purpose of this study was to investigate understand whether children with ADHD pay attention to essential cues when recognizing dynamic emotional facial expressions and whether these are associated with ToM and ADHD symptoms. The study findings revealed that children with ADHD possess deficits in the recognition of negative emotional facial expressions and in ToM skills. Children with ADHD paid less attention to the eye region, one of the areas of interest in the face for emotion recognition. Only the recognition of sad facial expressions was associated with ADHD symptoms. No relationship was observed between attention to facial areas of interest and ADHD symptoms or ToM tests. This study provides evidence that children with ADHD possess deficits in visual attendance to emotion cues. However, it also suggests that there is a separate area of social cognition that develops independently from the core symptoms of facial emotion recognition deficits in children with ADHD.

Studies investigating facial emotion recognition in ADHD patients have mostly reported deficits. These were most commonly observed in the fear facial expression [10,32–34]. However, studies have also reported deficits in the recognition of angry [35,36], sad [10,36], happy [35], disgusted [11,37], and neutral [38] facial expressions in individuals with ADHD. Other studies have reported no deficits in facial emotion recognition in ADHD [39–41]. Only a limited number of studies have used dynamic facial expressions to evaluate emotion recognition performance in children and adults with ADHD, and these have reported contradictory findings. Schwenck et al. found no deficits in facial emotion recognition, whereas an adult study reported deficits in the recognition of fearful and sad facial expressions [10,39]. Facial emotion recognition has been shown to be affected by the assessment tool employed, age, comorbid conditions, and medication use [8,9,42]. The ADHD group in this study being newly diagnosed with ADHD, not yet having started treatment, and not taking medication, the fact that primary school-age children were included, and disorders that would affect emotional recognition, such as specific learning disabilities and conduct disorder, being excluded are important in terms of revealing the effect of facial emotion recognition deficiencies in a pure ADHD group. A deficit was observed in the recognition of angry and disgusted facial expressions in the present study. The inability to recognize threatening emotions can cause significant problems in social learning [43].

ToM is thought to have evolved through the perceptual processing of the human face. The face is highly expressive because it emits a variety of distinctive cues that express internal biological states (such as happiness, fear, and anxiety) and that others can employ to make appropriate attributions [44]. Studies have shown that not focusing on the eye area of the face leads to poorer recognition of emotions [45]. A recent study found that individuals with ADHD pay less attention to the eye region of human faces [13]. Individuals with ADHD are thought to receive more information from other parts of the face (such the mouth) in terms of facial emotion recognition [46]. The present study found that the ADHD group viewed the eye region of the face less on all images except for fear. However, only the facial expressions of anger and disgust were less recognized. The rate at which the mouth region of the face was examined for emotional expressions in the ADHD group was similar to that in the control group. Individuals with ADHD may activate compensatory mechanisms by using other areas of the face when recognizing emotions. More cognitive strategies are required when distinguishing more complex emotions, such as anger and disgust, during emotional perception [47]. The lack of recognition of emotional facial expressions of anger and disgust may be explained in terms of inadequate gaze in the eye area of the face. Moreover, deficits in ToM skills may be related to difficulties in the recognition of these emotions. However, only the recognition of disgust and ToM skills being positively correlated in the ADHD group in this study, and not the other emotions, may indicate deficits in these two social cognition skills in different areas.

Research suggests that inattention and impulsivity lead to emotion recognition deficits in children with ADHD [48,49]. It has been suggested that children with ADHD not only experience difficulties recognizing emotions, but that attention problems also affect emotion recognition based on contextual information [50]. On the other hand, children with the hyperactive/impulsive subtype of ADHD have been reported to make more errors in recognizing emotional facial expressions than controls [42]. Another previous study also reported no difference in recognition of emotional expressions between ADHD subtypes [51]. Katz-Gold et al. demonstrated that emotional processing deficits in children with ADHD are unrelated to fundamental cognitive problems, such as impulsivity and inattention, affecting ADHD subtypes [52]. A recent study found no relationship between emotion recognition deficits and inattention and impulsivity measured by continuous performance tasks in children with ADHD [53]. Sjowall et al. demonstrated that emotion regulation and recognition exert separate effects in ADHD independent of cognitive abnormalities [54]. In the present study, the lack of correlation between ADHD symptoms and facial emotion recognition (except for sadness) and viewing areas of interest casts doubt on the effect of cardinal ADHD symptoms on emotion recognition. Children with emotion recognition deficits in ADHD can be considered a separate ADHD subgroup based on neurocognitive profiles [55]. Less focus on the eye region of the face among individuals with ADHD can be evaluated in this context. A positive correlation has been observed between sad facial expressions and symptom subscales in children with ADHD. Such children have more negative experiences and feel and display stronger emotional reactions in the face of these [56]. In other words, it may be suggested that children with high ADHD severity are more likely to encounter sad events and are better at recognizing this emotion. However, it is worth noting that of the negative emotions, only sadness was associated with ADHD symptoms, and not anger or disgust. The low correlation between a sad facial expression and symptom subscales and the absence of any correlation with other facial expressions in this study suggest that emotional facial recognition deficits are independent of ADHD symptoms. Emotion recognition deficits in ADHD can therefore be regarded as a separate difficulty that should be evaluated and treated together with ToM deficits.

Interventions in social cognition, in addition to treatment of ADHD, improve children’s functioning because impairments in social cognition in ADHD have adverse consequences in terms of social competence [2,57]. The lower level of concentration on the eye region, one of the most interesting areas of the face, and the poorer ToM skills observed in the ADHD group are essential for interventions. Developmental delays in social cognition in children with ADHD may be caused by reduced attention to the region of interest of emotional expression. It may therefore be helpful to focus the attention of children with ADHD on important facial regions during interventions. Social cognition interventions for children with ADHD may reduce social problems by improving higher-order ToM skills. Future research should investigate whether these interventions can help with the social problems of children with ADHD.

The particular strengths of this study include the use of a method with high ecological validity using dynamic emotional facial expressions, and the combination of eye-tracking technology with a ToM measure. Other strengths are, in contrast to other studies, the exclusion of specific learning disability comorbidity [58], which may cause difficulties in emotional facial recognition, the exclusion of all comorbidities in order to reduce confounding, and the selection of individuals with ADHD who were not taking medication in order to observe the effect of the disease.

However, the results should also be interpreted in light of the study’s limitations. In particular, the small sample size limited its statistical power. The fact that ADHD subtypes were not equally distributed may also have affected the results. Further studies are now needed due to the high prevalence of comorbid disorders in ADHD and heterogeneity in developmental pathways [59]. In addition, since this study involved children with ADHD, its validity in adolescents and adults is limited. Accurately identifying emotions in real-life interactions is dependent on the social cues displayed. The context in which an emotional facial expression is displayed has been shown to influence eye-gaze patterns [60]. Future research should investigate whether a deficit exists in the areas of interest of the face in social situations using the eye-tracking method and the relationship with social cognition.

Studies of emotion recognition difficulties in individuals with ADHD have generally focused on hot cognition, such as facial emotion recognition and ToM. Face recognition processes are also related to neuropsychological aspects unrelated to emotional content, such as memory and recall [8]. Future studies might usefully investigate the relationship between deficits in facial emotion recognition and other cognitive disorders in individuals with ADHD. Electrophysiological studies may shed light on this by showing which perceptual stages are involved in the recognition of facial expressions.

This study provides evidence that children with ADHD experience deficits in visual attention to emotion cues. However, it also suggests that there is a separate area of social cognition that develops independently from the core symptoms of facial emotion recognition deficits in children with ADHD. The ability to accurately perceive the emotions of those with whom we interact is very important for the effective regulation of social behavior. Understanding and treating the social difficulties of individuals with ADHD may improve their social functioning. Further studies with larger numbers of participants assessing the effects of impairments in social cognition will provide important information about facial emotion recognition deficits in individuals with ADHD.
